# Psychological flexibility in the work-caregiving interplay of employed family caregivers of people with dementia (DAILY-FLEX): Protocol of an experience sampling study

**DOI:** 10.1371/journal.pone.0361760

**Published:** 2026-10-30

**Authors:** Nahal Amini Moghaddam, Sara Laureen Bartels, Michaela Schok, Arnold B. Bakker, Golnaz Atefi, Marine Markaryan, Marjolein de Vugt

**Affiliations:** 1 Department of Psychiatry and Neuropsychology and Alzheimer Centrum Limburg, Mental Health and Neuroscience Research Institute, Maastricht University, Maastricht, The Netherlands; 2 Department of Clinical Neuroscience, Karolinska Institutet, Stockholm, Sweden; 3 Department of Clinical Psychology, Utrecht University, Utrecht, The Netherlands; 4 Center of Excellence for Positive Organizational Psychology, Erasmus University, Rotterdam, The Netherlands; 5 School of Economics and Business, University of Ljubljana, Ljubljana, Slovenia; 6 School of Management, Human Resource Management, University of Vaasa, Vaasa, Finland; 7 Department of Psychology, Lingnan University, Tuen Mun, Hong Kong; 8 Department of Industrial Psychology and People Management, University of Johannesburg, Johannesburg, South Africa; 9 Radboud University Medical Centre, IQ Health, Nijmegen, The Netherlands; 10 Radboud Alzheimer Centre, Nijmegen, The Netherlands; STIKES Wira Medika PPNI Bali: Sekolah Tinggi Ilmu Kesehatan Wira Medika PPNI Bali, INDONESIA

## Abstract

**Background:**

Employed family caregivers of people with dementia face competing work and caregiving demands that can negatively affect well-being across both domains. The Work–Home Resources model proposes that demands and resources influence cross-domain functioning through changes in personal resources. Psychological flexibility (PF), the ability to remain open to difficult experiences while acting in line with personal values, may represent a dynamic personal resource in this process. However, its real-time role in the daily work–caregiving interface remains largely unknown.

**Objective:**

This protocol describes the DAILY-FLEX study, which aims to investigate momentary and daily bidirectional spillover between work and caregiving demands/resources and domain-specific well-being, and to examine whether PF mediates these associations.

**Methods:**

This observational intensive longitudinal study uses Experience Sampling Methodology combined with baseline assessment. A minimum of 30 employed family caregivers of people with dementia will be recruited in the Netherlands through memory clinics, caregiver networks, and community outreach. Participants will complete a baseline questionnaire followed by a 14-day ESM protocol consisting of eight semi-random momentary assessments and one evening questionnaire per day using the m-Path smartphone application. Momentary assessments capture contextual demands and resources, PF, caregiving-related affect, and work engagement. Evening questionnaires assess daily work and caregiving demands/resources, PF, general well-being, and daily job satisfaction. Multilevel mixed-effects models and longitudinal mediation analyses will be used to examine lagged cross-domain spillover and PF as a within-person mediator.

**Discussion:**

This study provides a fine-grained examination of how work and caregiving interact in daily life among employed family caregivers of people with dementia. By focusing on PF as a dynamic mediating mechanism, the study may identify actionable targets for tailored psychosocial interventions and workplace support strategies aimed at improving caregiver well-being and sustainable employment.

## Introduction

Dementia is a major global health challenge, with a significant impact on affected individuals, their families, and society as a whole [1]. As the global population ages, the prevalence of dementia is expected to rise dramatically. The World Health Organization (WHO) reports that around 55 million people globally are currently living with dementia, a figure projected to rise to about 78 million by 2030 and 139 million by 2050 [2]. Informal caregivers, typically family members such as children or spouses, provide the majority of care for people with dementia, enabling them to live in their own homes for as long as possible [3]. Family caregivers perform essential roles, including personal care, task supervision, and assistance with daily activities, often without financial compensation [4]. Providing care for people with dementia can be perceived as rewarding and meaningful [4]; however, it is also demanding and may present challenges to the personal and professional lives of caregivers, especially when they are employed [4]. Recent research indicates that especially younger family caregivers (under 65) often continue working while providing care [4]. European studies suggest that approximately one-third to nearly half of family caregivers of people with dementia are in paid employment [5–7]. In the United States, nearly 60% of family caregivers of people with dementia are employed [3] compared with approximately one quarter in Australia [8].

Research in the context of dementia caregiving shows that juggling professional responsibilities with the emotional and physical demands of caregiving can lead to role overload, chronic stress, and emotional exhaustion [4,9–11]. Employed family caregivers often experience guilt, time pressure, and constant worry about meeting work and care demands, which can lead to sleep problems, anxiety, depression, and reduced life satisfaction [12,13]. While employment can provide structure, social connection, and a sense of identity beyond caregiving, balancing both roles depends heavily on external support, resources, and conditions outside the caregiver’s personal control (e.g., workplace flexibility and support from colleagues); without these, the combined demands can significantly harm caregivers’ mental and physical health [14,15]. Despite high demands, some caregivers report positive outcomes from managing both roles, including high levels of work performance, increased resilience to stress, and the opportunity for temporary relief from care duties while at work [4]. In the Netherlands, caregiving for a family member with dementia has a significant impact on employment. According to the Dementiamonitor in 2024, 20% of employed caregivers reported reducing their working hours due to caregiving responsibilities, with an average reduction of 12 hours per week (15 hours for men and 11 hours for women) [7]. Moreover, 74% report difficulties in balancing work and caregiving, underlining the substantial impact of dementia care on employment [7].

Supporting the quality of life and work–life balance of employed family caregivers is essential, not only for the family caregivers’ well-being but also for sustaining care systems. Family caregivers enable individuals with dementia to remain living at home for longer, delaying institutionalization, and reducing healthcare costs [3,16]. The contributions of family caregivers ease the burden on formal care systems [17,18], but prolonged strain can undermine their ability to provide care [19]. Therefore, understanding what impacts caregivers’ well-being is critical to identify needs and develop interventions that support both family caregivers and care recipients [20].

### Work–Home Resources model in the work–caregiving interplay

Balancing paid employment with caregiving for a person with dementia can place substantial strain on family caregivers’ well-being. To understand how these dual roles interact, the Work–Home Resources (W-HR) model provides a useful theoretical framework [21] (Fig 1). The model explains how experiences in one life domain, work or the home domain, in which informal caregiving occurs, influence functioning in the other domain through gains or losses in volatile personal resources such as time, energy, and positive affect. Accordingly, demands and resources arising from paid work and dementia caregiving may generate cross-domain spillover effects that shape caregivers’ well-being and functioning.

**Fig 1:**
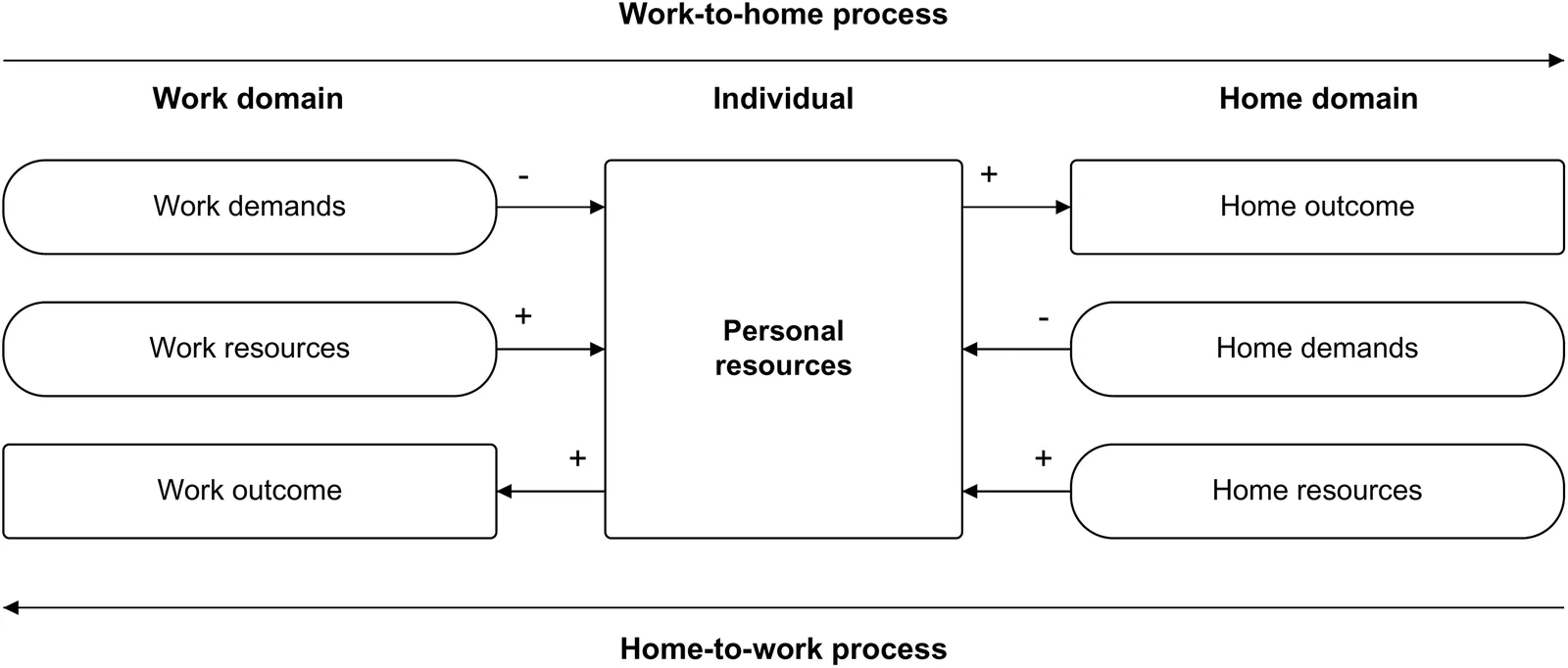
The Work–Home Resources model. Adapted from ten Brummelhuis and Bakker [21]. The model illustrates how contextual demands and resources influence personal resources, which in turn affect functioning and well-being across the work and caregiving domains.

Within the W-HR framework, contextual demands refer to physical, emotional, or cognitive stressors in the environment that require sustained effort and may deplete personal resources. In contrast, contextual resources are supportive aspects of the environment that help individuals cope with demands, fuel personal resources, and maintain functioning. For family caregivers of people with dementia, contextual demands may include intensive caregiving tasks, managing neuropsychiatric symptoms, and balancing caregiving with work responsibilities. As dementia progresses, increasing cognitive and functional impairments often lead to greater caregiving demands and more complex behavioral management, which are associated with poorer caregiver well-being and higher levels of depression and anxiety [22–25]. Conversely, contextual resources may include support from family members, access to formal care services such as respite care, or work-related autonomy and social support [3,21].

According to the W-HR model, these contextual conditions influence outcomes in the other life domain through their impact on personal resources. Personal resources may include daily energy, positive affect, resilience, or adaptive coping capacities. For employed caregivers of people with dementia, high demands in both work and caregiving domains combined with limited resources may accelerate personal resource depletion and intensify cross-domain strain [4,21]. In contrast, sufficient contextual and personal resources may help sustain functioning and well-being across life domains. Importantly, the W-HR model links these resource processes to a range of outcomes, including performance-related outcomes, behavioral outcomes such as withdrawal or role disengagement, and attitudinal outcomes such as satisfaction, emotional responses, and psychological well-being [21].

Combining employment and caregiving can generate both negative and positive cross-domain outcomes, commonly conceptualized as work–family conflict (WFC) and work–family enrichment, respectively [26]. WFC occurs when demands in one domain interfere with participation in the other [27], whereas work–family enrichment reflects situations in which experiences in one role enhance functioning in the other [28]. Among employed family caregivers of people with dementia, WFC arises both when work demands interfere with caregiving responsibilities and when caregiving demands hinder work performance [4,14,15,26,29]. Consistent with this bidirectional strain, these caregivers report higher levels of WFC, as well as greater absenteeism and presenteeism, and poorer physical and psychological well-being compared with non-caregivers [4,30–33]. Conversely, enrichment can occur when resources gained in one domain support functioning in the other [29]. Together, these processes highlight the dynamic interplay between work and caregiving roles and underscore the importance of examining both contextual and key personal resources within the W-HR framework [21].

### Psychological flexibility (PF) as a personal resource

Within the W-HR framework, personal resources play a central role in linking contextual conditions to outcomes. These resources can be differentiated by their temporal stability. Volatile, state-like resources – such as momentary positive affect, time, and energy – fluctuate within individuals and reflect their immediate capacity to act. In contrast, more stable, trait-like resources – such as resilience or general coping styles – represent enduring capacities that help individuals manage demands, mobilize and utilize contextual resources, and sustain well-being across situations [21,34].

In the present study, PF is conceptualized as a dynamic, volatile personal resource that fluctuates across moments and days and operates as a mediating mechanism in the work–caregiving interface. PF refers to the ability to remain open to internal experiences, maintain present-moment awareness, and act in accordance with personal values [35]. Rather than being treated as a fixed individual characteristic, it is understood here as a context-sensitive capacity that varies in response to situational demands and available resources. This capacity enables individuals to respond adaptively to stressors, utilize available resources effectively, and prevent resource loss across life domains [21,36].

PF is grounded in Acceptance and Commitment Therapy (ACT), a process-based behavioral approach that focuses on how individuals relate to internal experiences and engage in valued actions despite difficulties [35,37]. This approach is particularly relevant in dementia caregiving, where individuals frequently face emotionally challenging and often uncontrollable situations. In such contexts, momentary fluctuations in PF may determine whether caregivers become overwhelmed by distressing thoughts and emotions or are able to respond in an adaptive, value-consistent manner. Thus, PF reflects an immediate regulatory capacity through which caregivers manage both internal experiences (e.g., guilt, intrusive thoughts) and external demands [38]. It also supports engagement in value-based actions, such as maintaining social connections, pursuing meaningful activities, and providing care to loved ones [39].

The PF construct is theoretically described by the ACT Hexaflex model, which comprises six interrelated processes: acceptance, defusion, present-moment awareness, self-as-context, values, and committed action [35,37]. To capture their functional overlap more parsimoniously, the ACT Triflex model organizes these processes into three domains: *open up*, *be present*, and *do what matters* [40,41]. In the present study, the Hexaflex provides the theoretical foundation, while the Triflex informs the operationalization of PF in daily life (Fig 2). *Open up (i.e., acceptance and defusion)* means making space for unwanted thoughts and feelings instead of struggling with them, while learning to see thoughts as just thoughts rather than absolute truths or commands. It reduces avoidance and loosens the grip of rigid thinking, fostering openness to inner experiences [37]. *Be Present (i.e., Present-Moment awareness & Self-as-Context)* involves cultivating mindful awareness of both internal experiences (thoughts, feelings) and the external environment as they unfold in the here and now, while maintaining a flexible perspective of the self as the observer. It enhances clarity, perspective-taking, and connection with others [37]. *Do what matters (i.e., Values & Committed Action)* refers to identifying deeply held personal values and persistently taking action guided by those values, even in the face of obstacles [37].

**Fig 2:**
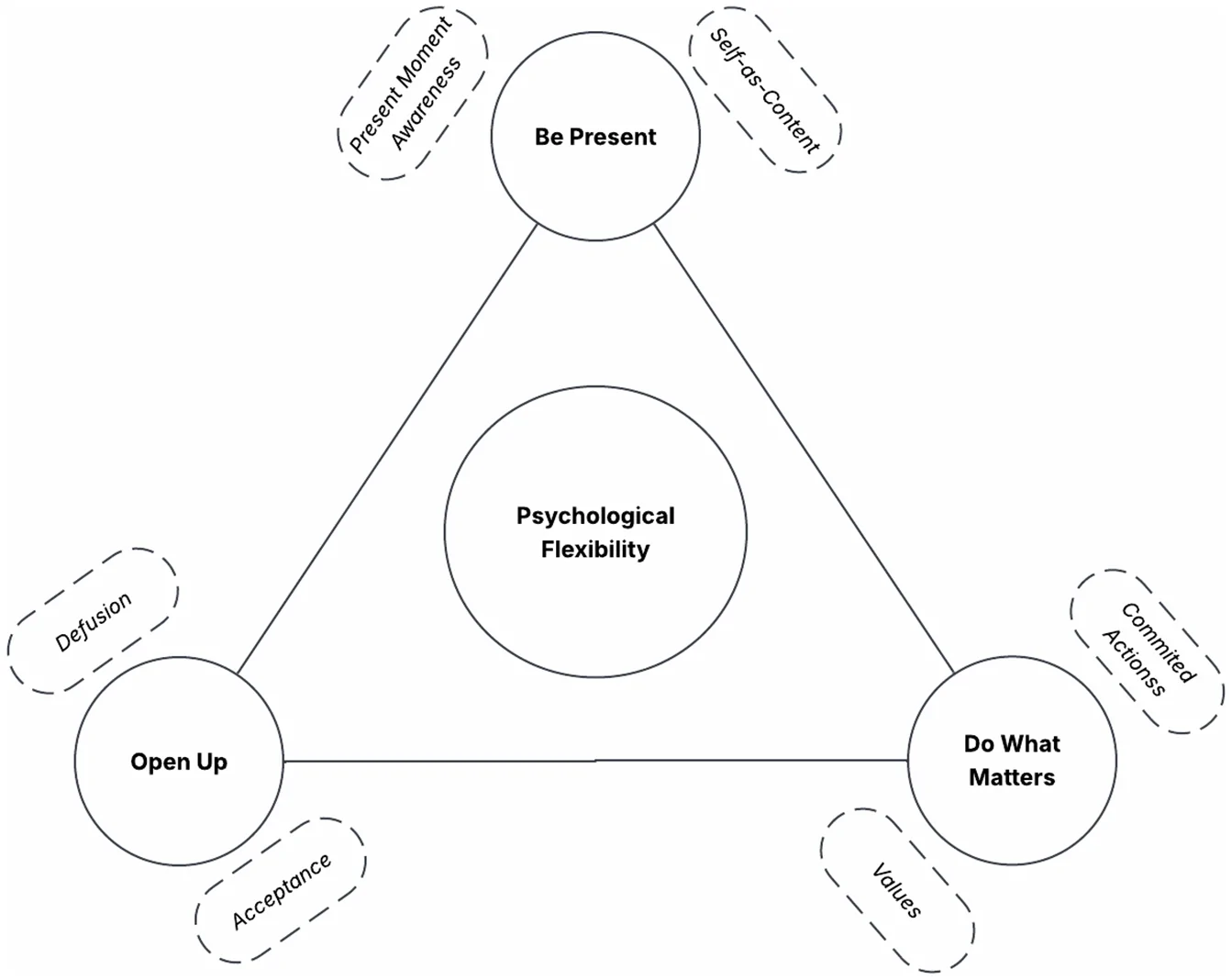
The Triflex model of PF. The model comprises the domains *Open Up*, *Be Present*, and *Do What Matters*, which integrate the six core ACT processes. Conceptually adapted from Harris, 2009, p. 15 [40].

From the perspective of the W–HR model, PF operates as a proximal, within-person mediator that transmits the effects of contextual demands and resources on well-being across domains [21,42]. Specifically, situational demands and resources in one domain (e.g., work or caregiving) are expected to influence momentary levels of PF, which in turn shape subsequent functioning and well-being in the other domain. This assumption is consistent with resource-based theories of stress, which posit that demands consume cognitive and emotional resources, whereas resources facilitate adaptive regulation and functioning [43,44]. In line with process-based accounts of PF, this capacity reflects individuals’ momentary ability to regulate internal experiences and maintain value-consistent action under varying contextual conditions [35,45]. For instance, high demands may reduce PF by increasing cognitive and emotional load, thereby limiting individuals’ capacity to regulate internal experiences and act in a value-consistent manner [43,46]. In contrast, the availability of contextual resources (e.g., autonomy, social support) may enhance PF by facilitating adaptive regulation, engagement, and goal-directed behavior [41,42].

Through this mechanism, PF translates contextual conditions into downstream outcomes. When PF is reduced, individuals are more likely to become entangled with distressing thoughts and emotions, increasing the likelihood that demands spill over into strain and impaired functioning in the other domain [35,45]. Conversely, when PF is enhanced, individuals are better able to maintain adaptive responding, sustain engagement, and utilize available resources effectively, thereby promoting positive spillover and well-being [28,41]. In this way, PF represents a key explanatory mechanistic process through which both negative (resource loss) and positive (resource gain) spillover effects unfold in daily life.

Evidence from dementia caregiving research supports this hypothesized mechanism. Psychological inflexibility has been associated with higher levels of depression, anxiety, and caregiver burden, indicating that difficulties in regulating internal experiences amplify the impact of caregiving demands [47,48]. Conversely, greater PF has been linked to better mental health, higher life satisfaction, and more adaptive coping among caregivers of people with dementia [38,39,49]. Taken together, these findings are consistent with the assumption that PF functions as a dynamic mechanism through which daily demands and resources are translated into well-being outcomes, rather than merely buffering or strengthening these relationships.

### Present study and aim

Understanding the dynamic interplay between work and caregiving, and in particular the mechanisms through which these domains influence each other, is essential for developing tailored psychosocial interventions for employed family caregivers of people with dementia. Existing caregiver research remains limited in its focus on positive experiences [50] and often relies on retrospective self-reports and cross-sectional designs [51]. Such approaches may produce memory-biased accounts of daily life, as individuals tend to over- or under-report past experiences [52,53], while overlooking the moment-to-moment fluctuations in demands, resources, and well-being that characterize everyday caregiving [54]. These fluctuations are particularly relevant in dementia caregiving, as symptoms of people with dementia vary across and within days, leading to changing care demands [55]. Moreover, PF is considered context-dependent and may also vary across daily situations [41]. Therefore, studying caregiver experiences in real time is crucial to better understand the daily dynamics of the work–caregiving interface.

Intensive longitudinal sampling (ILS), including the Experience Sampling Method (ESM), provides a powerful approach to capture these everyday experiences. ESM involves repeated within-person assessments across short periods (e.g., several times per day for multiple consecutive days), typically through smartphone-based diaries, allowing researchers to examine dynamic processes as they occur in natural environments while reducing recall bias [56]. A recent scoping review identified 48 ILS studies involving family caregivers of people with dementia, demonstrating the feasibility of such approaches in this population [51]. However, only one study has specifically examined employment-related factors in dementia caregivers, showing that employment status moderates associations between sleep quality, caregiving burden, and negative affect [57]. Importantly, prior work has not investigated the real-time work–caregiving interface, the role of PF as a dynamic process, or the short-term within-person mechanisms through which cross-domain effects unfold.

To address these gaps, the present study applies an ILS design using ESM to investigate the real-time interplay between work and caregiving among employed family caregivers of people with dementia, with a specific focus on PF as a dynamic, within-person personal resource that mediates cross-domain processes. The present study is part of the broader DAILY-FLEX project, a coordinated research initiative examining the role of PF in caregiver well-being using a shared ESM dataset.

The study examines how momentary and daily experiences in one domain (work or caregiving) influence subsequent functioning and well-being in the other domain through changes in PF. The primary outcomes of interest are domain-specific indicators of well-being and functioning. At the momentary level, well-being is conceptualized as domain-specific, including caregiving-related well-being (momentary positive and negative affect during caregiving episodes) and work-related well-being, operationalized as momentary work engagement during work episodes. Work engagement is selected as a key indicator of functioning, as it reflects the extent to which individuals are able to remain energized, involved, and effective in their work [58]. At the daily level, outcomes focus on more general evaluative indicators of functioning across domains. Specifically, daily well-being reflects overall affective and energy-related states, whereas daily work-related well-being is operationalized as daily job satisfaction, capturing individuals’ overall evaluation of how their workday went. This approach aligns with the W–HR model, which emphasizes attitudinal outcomes (e.g., well-being, engagement, satisfaction) as proximal indicators of successful adaptation to cross-domain demands before longer-term consequences such as task performance emerge.

Guided by the W–HR model, the study tests whether demands and resources in one domain (work or caregiving) predict subsequent well-being in the other domain, and whether these cross-domain effects are transmitted through momentary and daily fluctuations in PF. Specifically, demands are expected to reduce PF, thereby increasing the likelihood of negative spillover, whereas resources are expected to enhance PF, thereby facilitating positive spillover and improved well-being. By integrating momentary and daily perspectives, the study provides a fine-grained understanding of how cross-domain dynamics unfold in everyday life and identifies PF as a key explanatory mechanism underlying the work–caregiving interface.

### Research questions and hypotheses

The following research questions (RQ) and hypotheses (H) will be addressed (see Figs 3 and 4). Spillover processes will be examined at both the momentary level (across consecutive experience sampling assessments) and the daily level (across consecutive days), allowing for both fine-grained and more aggregated perspectives on work–caregiving dynamics.

**Fig 3:**
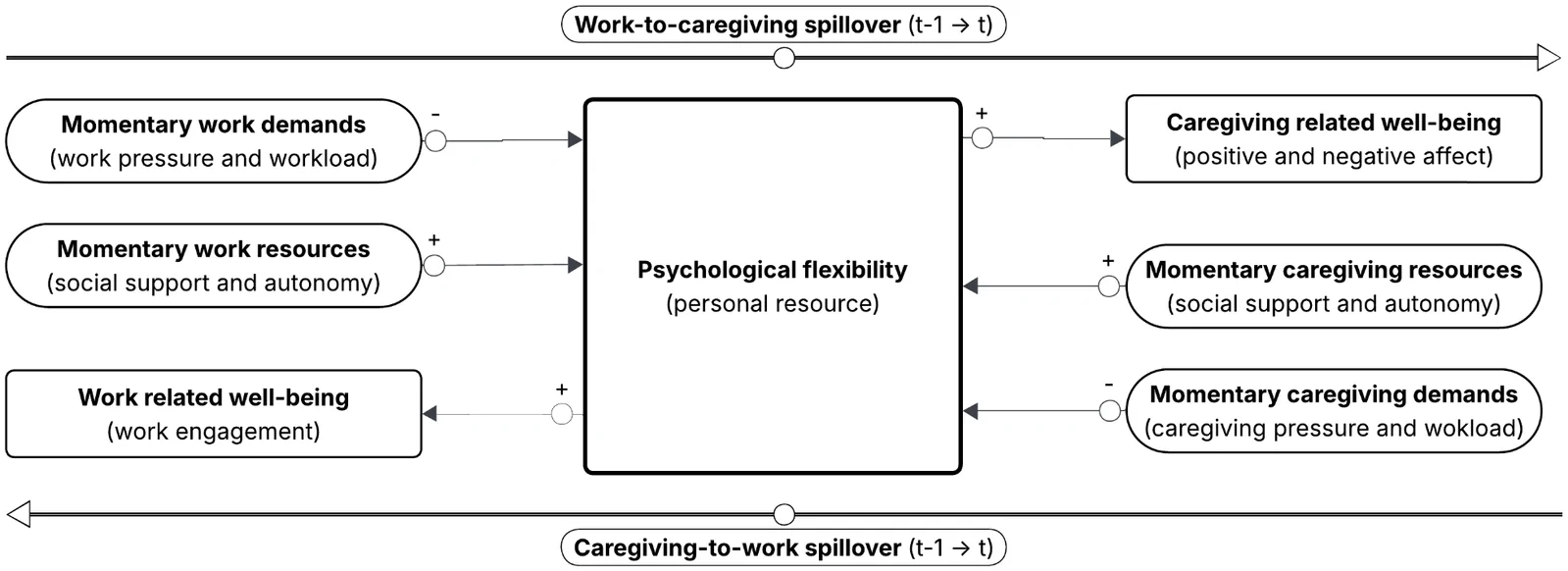
Momentary level hypotheses. Depicts the hypothesized momentary spillover processes from work-to-caregiving (H1) and caregiving-to-work (H2), with domain-specific well-being as outcomes. PF is modeled as a within-person mediator (H3), transmitting the effects of momentary demands and resources in one domain at time t − 1 on subsequent well-being in the other domain at time t.

**Fig 4:**
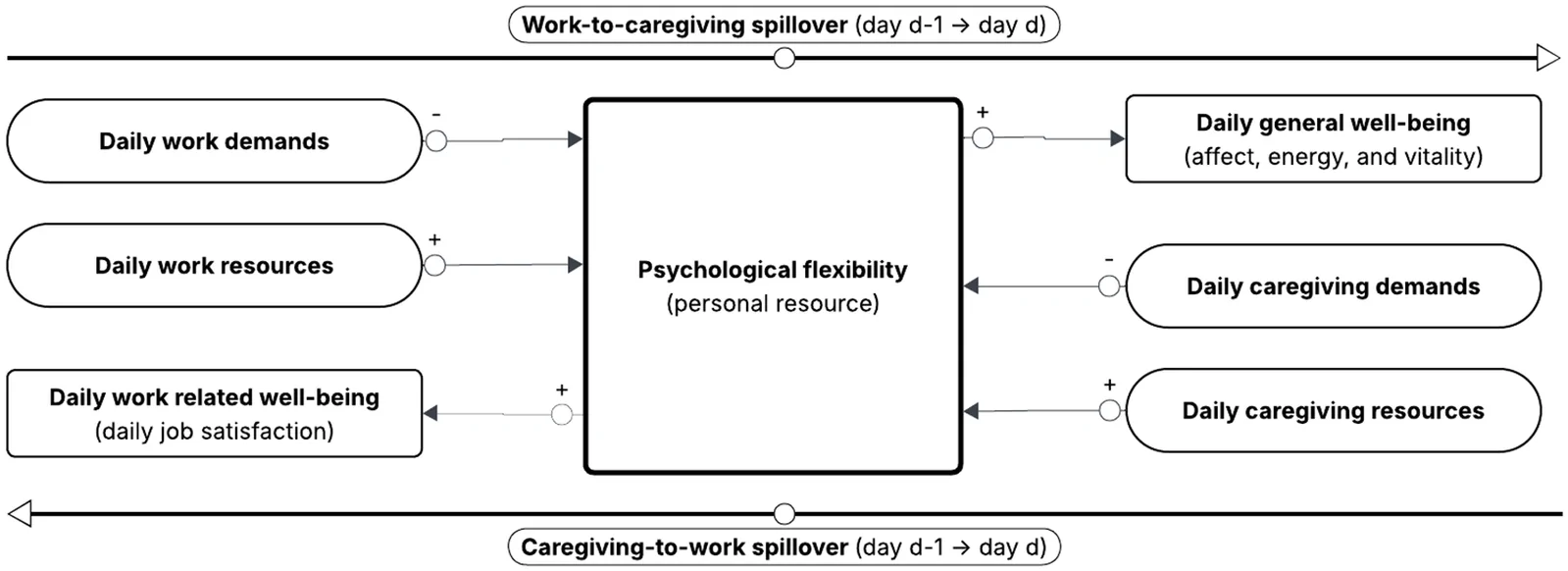
Daily level hypotheses. Illustrates the hypothesized daily spillover processes, in which daily work and caregiving demands and resources on day d − 1 predict subsequent daily well-being on day d (H1) and daily work-related well-being on day d (H2). PF on day d is modeled as a dynamic, within-person mediator (H3) of these associations.

**RQ1.** Do work-related and caregiving-related demands and resources, operationalized through specific momentary indicators (e.g., work pressure, workload, social support, autonomy, caregiving pressure, and caregiving workload), spill over across domains and influence subsequent domain-specific well-being at both the momentary and daily levels in employed family caregivers of people with dementia?

**H1. Work-to-caregiving spillover (momentary and daily).** At the momentary level, higher work-related demands (i.e., greater work pressure and workload) and lower work-related resources (i.e., lower perceived social support and autonomy) at a previous assessment (t − 1) will be associated with poorer caregiving-related well-being at the subsequent assessment (t), reflected in lower positive affect and higher negative affect during caregiving moments. At the daily level, higher work demands and lower work resources on day d − 1 will predict lower daily general well-being on day d.
**H2. Caregiving-to-work spillover (momentary and daily).** At the momentary level, higher caregiving-related demands (i.e., greater caregiving pressure and caregiving workload) and lower caregiving-related resources (e.g., lower perceived social support and autonomy in caregiving) at a previous assessment (t − 1) will be associated with poorer work-related well-being at the subsequent assessment (t), reflected in lower work engagement during work moments. At the daily level, higher caregiving demands and lower caregiving resources on day d − 1 will predict lower daily work-related well-being (i.e., daily job satisfaction) and lower daily general well-being on day d.

**RQ2.** Does PF mediate the bidirectional spillover associations between work and caregiving demands/resources and subsequent domain-specific well-being at the momentary and daily levels, respectively?

**H3. Mediation by PF.** PF will mediate the bidirectional spillover associations between demands/resources and cross-domain well-being. At the momentary level, demands and resources at the preceding assessment will predict subsequent PF and domain-specific well-being. At the daily level, demands and resources on day d − 1 will predict PF on day d, which will in turn be associated with well-being on day d.

## Materials and methods

### Study design

An observational, longitudinal study will be conducted using ESM. The study combines an intensive longitudinal component with a baseline assessment to comprehensively capture the work–caregiving interface in employed family caregivers of people with dementia. The reporting of this protocol was informed by recommendations for observational study protocol reporting [59]. To enhance transparency and reproducibility, the study protocol, research questions, hypotheses, and planned analytical strategy have been pre-registered on the Open Science Framework (OSF) prior to data analysis [60]. Any deviations from the pre-registered protocol, including exploratory or post-hoc analyses, will be clearly documented and justified in subsequent publications. This is an observational study and does not prospectively assign participants to any health-related intervention.

### Study status and timeline

Participant recruitment for the DAILY-FLEX study began in November 2025 and is ongoing at the time of manuscript submission. Recruitment is expected to continue until the target sample size has been achieved. Data collection is expected to be completed by January 2027, followed by data cleaning, statistical analyses, and dissemination of the primary findings. This manuscript describes the study protocol, which was finalized before completion of data collection and prior to analysis of the primary outcomes.

### Setting and recruitment

The study will be conducted among employed family caregivers of people with dementia. It is coordinated by researchers from Alzheimer Centrum Limburg (ACL), Department of Psychiatry and Neuropsychology, Maastricht University/Maastricht University Medical Centre+ in the Netherlands. A purposive sampling strategy will be used to recruit employed family caregivers [61]. Participants will be recruited through multiple channels to enhance reach and heterogeneity of the sample. Relevant sources of heterogeneity are expected to include caregiver sociodemographic characteristics, employment conditions (e.g., working hours and work arrangements), caregiving context (e.g., relationship to and cohabitation with the person with dementia, caregiving intensity, and availability of formal or informal support), and characteristics of the person with dementia (e.g., dementia type, disease duration, and neuropsychiatric symptom burden). These characteristics will be assessed at baseline to describe the sample and contextualize between-person differences in daily work–caregiving experiences. Recruitment will take place via the memory clinic of Maastricht University Medical Centre+, where clinicians will identify and inform eligible family caregivers, and communication channels of Alzheimer Nederland; through self-referral channels such as social media and caregiver support networks; and through community-based outreach, including regional case managers affiliated with Hulp bij Dementie Limburg and activities of ACL (e.g., client panel, Alzheimer Cafés). Data will be collected remotely in participants’ natural environments (home, workplace, and community) using a smartphone-based ESM application. Baseline questionnaires and informed consent will be completed online via secure platforms (see Baseline assessment section).

### Participants

#### Screening and enrollment.

Screening will be conducted through a QR code on the study flyer, which links potential participants to a secure REDCap platform. REDCap is a web-based application designed for creating and managing online databases and surveys, and it operates in compliance with the General Data Protection Regulation (GDPR) [62]. Interested individuals are asked to self-report their eligibility by confirming eligibility (see Inclusion and exclusion criteria section). Contact details (telephone number and email address) are collected through the same platform. A trained research assistant subsequently contacts each respondent to verify eligibility, answer questions, and provide information about study participation prior to informed consent.

Eligible participants who agree to participate will receive the Participant Information Sheet and provide written informed consent digitally through REDCap.

#### Inclusion and exclusion criteria.

Eligibility criteria were defined to ensure that participants were actively engaged in both employment and dementia caregiving during the study period. Eligible participants are individuals who meet all of the following conditions: they are the family caregiver of a person with dementia, are aged 18 years or older, are fluent in Dutch or English, are able and willing to use a smartphone for remote data collection, are currently employed, and are able to provide informed consent. Participants will be excluded if any of the following conditions apply: the presence of acute or severe comorbidity (e.g., psychotic disorder, suicidal risk), planned surgery during the study period, caring for a person with dementia residing in a care home, anticipated major situational changes within the next month (e.g., an extended holiday or transition of the care recipient into institutional care), anticipated inability to comply with the study procedure, or being currently on sick leave.

#### Sample size.

A minimum target of N = 30 employed family caregivers will be recruited for this exploratory ESM study. This target reflects a balance between methodological considerations and the feasibility of recruiting a hard-to-reach population of employed family caregivers of people with dementia. In addition, the study will remain open to recruiting more participants if feasible within the planned recruitment period of 14 months.

Sample size considerations in intensive longitudinal research differ from those in conventional cross-sectional designs because statistical power and estimation precision depend jointly on several features of the study design, including the number of participants, the number of repeated observations per participant, the magnitude of the effects of interest, and the variance and temporal dependency structure of the data [63–65]. In the present study, each participant can contribute up to 112 momentary assessments (eight assessments per day across 14 days), corresponding to a maximum of **3,360 momentary observations** across 30 participants. In addition, participants complete one evening questionnaire per day, resulting in up to 14 daily assessments per participant and a maximum of **420 daily observations** across 30 participants.

The number of observations available for analysis will be lower than these theoretical maxima because incomplete compliance is expected and the planned lagged analyses require consecutive valid assessments. For domain-specific analyses, the effective number of observations may be further reduced because relevant work or caregiving contexts will not necessarily occur at every assessment. Previous ESM research indicates that compliance varies considerably across populations and study designs [66]. Therefore, rather than assuming that all scheduled assessments will contribute to the analyses, the available number of observations will be determined based on the completed assessments and valid lagged pairs observed in the final dataset.

The dense repeated-measures design provides substantial Level-1 information for estimating the primary within-person temporal associations. However, a large number of repeated observations within individuals does not fully compensate for a limited number of participants at Level 2, particularly for estimating between-person effects, random-effect variability, and more complex multilevel models [64,65,67]. Accordingly, the minimum target of 30 participants should not be interpreted as providing equivalent precision for all planned analyses. The primary analyses will focus on the hypothesized within-person temporal associations between work and caregiving demands and resources, PF, and well-being. Multilevel mediation analyses and analyses involving between-person differences will be interpreted cautiously in light of the Level-2 sample size.

Recruitment of larger samples is particularly challenging in the present study because eligibility requires participants to combine paid employment with family caregiving for a person with dementia, while participation additionally involves intensive repeated assessments over 14 consecutive days. Previous research has demonstrated the feasibility of using intensive longitudinal methods among family caregivers of people with dementia across a range of study designs and samples [51]. The minimum target of 30 participants is therefore considered a feasible recruitment target for examining the primary within-person processes of interest, while acknowledging the limitations associated with the participant-level sample size.

If recruitment exceeds the minimum target, all eligible participants will be included, thereby increasing the precision of parameter estimates and strengthening the analysis of more complex and between-person effects. Overall, the sample-size rationale follows a design-based approach to intensive longitudinal research, recognizing that statistical power and estimation precision depend on the combination of participant number, repeated measurements, effect sizes, variance structure, and temporal dependencies rather than on a single participant-number threshold [63–65].

### Patient and public involvement

The study was developed in collaboration with the Client Panel of ACL**,** composed of people with dementia and family caregivers (N = 13). During an interactive 1-hour session, the panel reviewed and tested the digital diary app and ESM items, providing feedback on clarity, relevance, and participant burden. Their input led to several refinements, such as clearer wording and improved usability. Moreover, a former family caregiver of a person with young-onset dementia who combined employment with caregiving for 1.5 years functions as an advisor on this project. This advisor is being consulted regularly to ensure the study design and interpretation of findings align with the lived experience of family caregivers. The client panel will also be consulted again to support the interpretation of findings.

In addition, we sought insights from the ESM expert group (Open University Heerlen/ Maastricht University) of the Belgian-Dutch ESM Network. This research community specializes in intensive longitudinal methods and provided methodological feedback on item formulation, sampling schedules, and digital diary design. Furthermore, clinicians and researchers experienced in ACT were consulted to ensure that the items adequately captured PF (open up, do what matters, and be present) in a momentary and context-sensitive way. Their combined expertise ensured that the ESM items used in the present study are scientifically sound, clinically meaningful, and aligned with best practices in ecological momentary assessment.

### Study procedure

#### Baseline assessment.

Following the provision of informed consent, participants will complete a set of baseline assessments administered online via Castor EDC. Castor EDC is a secure, cloud-based platform widely used in academic and clinical research for collecting and managing study data [68]. It is fully GDPR-compliant, which ensures that personal data are processed lawfully, stored securely, and only used for specified research purposes.

In addition to the intensive longitudinal component, a baseline assessment is conducted prior to the ESM period. Completion of the baseline assessment will take approximately 40–50 minutes (see Table 1). The baseline assessment serves an exploratory purpose, providing a comprehensive characterization of participants’ demographic, work, caregiving, and clinical context, as well as relatively stable individual characteristics (e.g., general well-being, PF, caregiving and work context characteristics). These baseline data are used to describe the sample, inform the interpretation of ESM findings, and explore between-person differences and potential covariates.

**Table 1:** Baseline variables, measurement, and purpose.

Domain	Variable	Measurement/Instrument	Purpose
Sociodemographic characteristics (caregiver)	Age, Sex, Gender, Marital status, Education level, Employment status & work arrangement	Self-report questionnaire	Sample description and background characteristics
Caregiving context	Relationship to person with dementia, Living situation, Contact hours per week, Caregiving hours per week, Day care use, Other caregivers/support, Perseverance time	Self-report questionnaire	Descriptive characterization of caregiving context
Person with dementia (care recipient)	Age, Sex, Gender, Dementia diagnosis, Time since first symptoms & diagnosis	Caregiver report	Baseline disease-related burden
Neuropsychiatric symptoms (care recipient)	Behavioral and psychological symptoms	Neuropsychiatric Inventory Questionnaire (NPI-Q) [69,70]	Care recipient’s symptoms and caregiver distress
General well-being (caregiver)	Overall mental well-being	WHO-5 Well-being Index [71,72]	Baseline mental health status
Psychological Flexibility	Psychological Flexibility	Psy-Flex questionnaire [73,74]	Personal resource
Caregiving context characteristics	Caregiver burden and positive aspects	Caregiver Reaction Assessment (CRA) [75,76]	Caregiving context description
Work context characteristics	Psychosocial work environment	Copenhagen Psychosocial Questionnaire III (COPSOQ-III) [77,78]	Work context description
Work-home interaction	Work–home conflict and enrichment (negative and positive work–home and home–work interactions)	Survey Work–Home Interaction Nijmegen (SWING) [79]	Baseline characterization of work–home conflict and enrichment

**Demographics and contextual characteristics.** Demographic information will be collected from participating family caregivers, including age, sex, gender, marital status, and educational level. Work-related characteristics will include employment status (e.g., full-time, part-time, self-employed), work arrangement, shift type, and average weekly working hours. Care-related characteristics will include the caregiver’s relationship to the person with dementia, cohabitation status, average weekly contact hours, and average weekly caregiving hours. Information will also be collected on the use of formal care services (e.g., day care), the presence of additional caregivers or support (paid and unpaid), and their roles and time contribution. Moreover, to describe the care recipient, caregivers will report demographic and clinical information about the person with dementia, including age, sex, gender, type of dementia, estimated time since first symptoms, and time since formal diagnosis. These variables will be used to contextualize caregiving demands and to describe heterogeneity in disease progression within the sample.

Neuropsychiatric symptoms of the person with dementia. To assess neuropsychiatric symptoms in persons with dementia and the related caregiver distress, the Neuropsychiatric Inventory Questionnaire (NPI-Q), a shortened version of the Neuropsychiatric Inventory, will be employed [69]. The NPI-Q covers twelve domains: delusions, hallucinations, agitation/aggression, depression/dysphoria, anxiety, euphoria, apathy, disinhibition, irritability, aberrant motor behavior, nighttime behavioral disturbances, and appetite/eating changes. For each domain, caregivers respond to a screening item indicating the presence or absence of the symptom. If the symptom is present, its severity is rated on a scale from 1 (mild) to 3 (severe), resulting in a total score ranging from 0 to 36. In addition, caregiver distress is assessed for each domain on a scale from 0 (not distressing) to 5 (extremely distressing). The Dutch version of the NPI-Q has been validated and is considered a reliable assessment tool [70]. In the present study, NPI-Q severity and distress scores will be used to characterize disease-related burden at baseline and may be considered as covariates in specific analyses when neuropsychiatric symptom burden represents a plausible confounder of the association under investigation.

**General Well-being.** General mental well-being of caregivers at baseline will be assessed using the WHO**-**5 Well-Being Index [71]. The WHO-5 Well-Being Index is a short, self-reported measure of current mental well-being. It consists of five positively worded items related to mood, vitality, and general interests (e.g., “*I have felt cheerful and in good spirits*”). Respondents are asked to rate how often they experienced these feelings over the past two weeks using a 6-point Likert scale, ranging from 0 (“At no time”) to 5 (“All of the time”). The raw score ranges from 0 to 25 and is typically multiplied by 4 to give a percentage score ranging from 0 (worst possible well-being) to 100 (best possible well-being). The WHO-5 has demonstrated good psychometric properties across a variety of populations and settings, including high internal consistency and construct validity [80]. The WHO website provides access to the validated Dutch version of this index [72]. Baseline well-being is included to describe participants’ general mental health status at study entry, examine between-person differences, and may be considered as a covariate in specific analyses when it represents a plausible confounder of the association under investigation.

**Psychological flexibility.** PF at baseline will be measured using the Psy-Flex questionnaire [73], a brief and contextually sensitive instrument developed within the ACT framework. The Psy-Flex consists of six items (e.g., *“I engage thoroughly in things that are important, useful, or meaningful to me”*), each representing one of the six core ACT processes: acceptance, cognitive defusion, present-moment awareness, self-as-context, values, and committed action. Respondents rate each item on a 5-point Likert scale ranging from 1 (“very seldom true”) to 5 (“very often true”), with higher scores indicating greater PF. The Psy-Flex has demonstrated strong psychometric properties, including good internal consistency (α ≈ .91) and unidimensional factor structure. Its brevity and sensitivity to context make it well suited for use in both clinical and non-clinical populations. The Dutch version of the Psy-Flex was obtained through the authors and adheres to the structure and intent of the original instrument [74]. In the present study, baseline PF will be used to characterize participants’ general level of PF and to explore whether stable between-person differences in PF are associated with average levels of demands, resources, momentary PF, and well-being across the study period. Baseline PF may additionally be considered in sensitivity or covariate analyses where appropriate.

**Caregiving context characteristics.** The Caregiver Reaction Assessment (CRA) will be used to assess caregiving demands and resources from the perspective of family caregivers [75]. The CRA is a multidimensional self-report instrument designed to capture both the positive and negative aspects of the caregiving experience. It includes 21 items across five subscales: caregiver esteem, lack of family support, impact on finances, impact on schedule, and impact on health. For the purposes of the present study, the subscale on caregiver esteem will be conceptualized as reflecting caregiving resources, as it captures positive appraisals and personal gains associated with the caregiving role. In contrast, the remaining subscales (i.e., lack of family support, financial impact, schedule disruption, and impact on health) will be considered indicators of caregiving demands, as they reflect various stressors and burdens experienced by caregivers. Each item is rated on a 5-point Likert scale ranging from 1 (strongly disagree) to 5 (strongly agree), and subscale scores are calculated separately, with higher scores indicating a greater presence of the respective construct. The CRA has demonstrated good psychometric properties and has been widely used in caregiving research, including studies involving dementia caregivers. A Dutch validation study [76] confirmed that the CRA maintains adequate psychometric properties in a Dutch sample of informal caregivers: each subscale showed acceptable internal consistency (Cronbach’s α ≈ 0.62–0.83) and the five-dimensional structure was supported, indicating that the CRA reliably captures both negative burdens and positive aspects of caregiving in this context. Given the intensive longitudinal design and the sample size, these baseline measures will primarily serve descriptive and exploratory purposes, complementing the repeatedly assessed state-level caregiving demands and resources.

**Work context characteristics.** Trait work demands and resources at baseline will be assessed using the Copenhagen Psychosocial Questionnaire (COPSOQ-III) [77]. This questionnaire contains items that are grouped into validated scales that each measure distinct psychosocial dimensions such as quantitative demands, emotional demands, influence at work, role clarity, social support, and work-life conflict. Participants will respond to 32 items and responses are recorded using standardized Likert-type response options**.** For example, frequency-based items use scales ranging from *“Always”* to *“Never/hardly ever,”* while agreement or intensity-based items range from *“To a very large extent”* to *“To a very small extent.”* The Dutch and English versions of the questionnaire used in the present study have been validated and show satisfactory to strong internal consistency across most scales, supporting its use for both research and organizational development purposes [77,78]. The COPSOQ-III scale is included to describe participants’ general work context at baseline and to provide descriptive background information.

**Work–home conflict and enrichment.** Work–home conflict and enrichment at baseline will be assessed using the Survey Work–Home Interaction Nijmegen (SWING). The SWING is a psychometrically validated self-report instrument developed in the Netherlands to assess bidirectional interactions between the work and home domains [79]. It distinguishes between negative and positive interactions, thereby capturing both work–home conflict and work–home enrichment. The SWING consists of 22 items distributed across four subscales: negative work-to-home interference and negative home-to-work interference, reflecting work–home conflict, and positive work-to-home interaction and positive home-to-work interaction, reflecting work–home enrichment. Each item assesses the frequency of specific work–home interaction experiences and is rated on a 4-point Likert scale ranging from 1 *(“never”*) to 4 *(“always”*). The SWING was developed and validated using five independent Dutch samples comprising a total of 2,472 participants and demonstrated a robust four-factor structure and good internal consistency across subscales. In addition, the instrument showed meaningful associations with external variables such as job characteristics, home environment factors, and indicators of health and well-being, supporting its construct validity in Dutch samples [79]. In the present study, the SWING is included to provide a general descriptive indication of participants’ perceived work–home interface at baseline.

#### The ESM briefing.

To capture participants’ real-time experiences and contextual factors throughout their daily lives, we will employ the ESM using a smartphone app. Apps like m-Path enable the scheduling and distribution of self-report questionnaires via participants’ smartphones, ensuring high temporal resolution of data collection in naturalistic settings [81].

To maximize participant compliance during the ESM protocol, several strategies will be implemented based on prior empirical evidence. First, to reduce the likelihood of missed responses due to low alertness or competing obligations, prompts will be scheduled after 8 am. Second, the sampling window will be tailored to each participant’s daily routine, allowing flexible adjustment of signal start times to align with individual caregiving and work schedules. Finally, participants will receive a briefing session to introduce the study procedures and the smartphone app, followed by ongoing contact through two check-in phone calls and availability of support throughout the data collection period. These measures are intended to promote engagement and ensure high-quality data collection [82]. Participants will be instructed on how to install the app on their mobile devices and complete the diary during a 30–45 min briefing session (phone call or in person, based on preference). Participants who do not have their own smartphone will receive one and will be trained on how to use it. A demo questionnaire will be presented to caregivers and an app manual containing all relevant information regarding the use of the smartphone app will be handed out afterwards (on paper or digitally, based on preference).

#### The ESM procedure.

After the briefing session, participants will be enrolled in a 14-day ESM protocol consisting of nine daily measurement points: eight momentary assessments and one end-of-day questionnaire. Assessments will be delivered via the m-Path smartphone app [81] at semi-random intervals between 08:30 and 22:00, with prompts distributed across the day to capture morning, midday, afternoon, and evening experiences. Each prompt directs participants to complete a short questionnaire lasting approximately 2–3 minutes. At each momentary assessment, participants report on their current context (e.g., activity and location), state work and caregiving demands and resources, momentary PF, and well-being. Responses are categorized into work contexts and caregiving contexts based on reported location, the nature of the activity (e.g., working vs. caregiving), and whether the activity involves the person with dementia. Momentary items include 7-point Likert-scale items (1 = not at all to 7 = very much), multiple-choice items, and yes/no questions (see S1 and S2 Appendices for the full item list). Compliance will be supported through brief automatic in-app reminders and regular contact with the research team, including check-ins on day 2 and day 7 of the ESM period.

#### Momentary ESM measures.

Momentary items will be administered multiple times per day to capture state-level experiences in both work and caregiving contexts (see Table 2). Each assessment will include contextualized items assessing demands, resources, well-being, and PF within the moment. In line with the across-domain hypotheses, variables measured in one domain will be used to predict outcomes in the other domain.

**Table 2:** Momentary ESM items.

Variable	Role	Related momentary item(s)
Activity pressure	Predictor	I have to work fast
Activity workload	Predictor	I have a lot to do
Social support	Predictor	I feel supported in doing this
Autonomy	Predictor	I can decide how to carry this out
Psychological Flexibility	Mediator	I let my thoughts and feelings come and go without controlling them I stay focused on what matters and engage in it I act like the sort of person I want to be
Positive Affect	Outcome	I feel cheerful I feel relaxed I feel enthusiastic I feel satisfied
Negative Affect	Outcome	I feel anxious I feel irritated I feel sad I feel tense I feel guilty I feel tired I feel lonely
Work Engagement	Outcome	I feel energetic I am proud of myself for doing this I am fully involved in doing this

**Predictors: Momentary demands and resources.** When participants indicate that they are currently engaged in work- or caregiving-related activities, they will be prompted to rate specific momentary demands and resources. These items are adapted from established retrospective measures within the Job Demands–Resources questionnaire [83] and reformulated to fit the momentary assessment context and capture state-level fluctuations in daily life. For example, demands will be assessed using items such as *“I have to work fast*” and “*I have a lot to do,*” reflecting perceived activity pressure and workload. Resources will be assessed using items such as “*I feel supported in doing this*” and “*I can decide how to carry this out*,” capturing perceived social support and autonomy. In line with the theoretical framing of the W–HR model [21], these indicators represent contextual demands and resources; specifically, activity pressure and workload reflect momentary demands, whereas perceived social support and autonomy reflect momentary resources. Because ESM focuses on concrete, context-specific experiences, emphasis is placed on these individual indicators rather than assuming a uniform higher-order demands/resources construct across all participants. This approach aligns with idiographic perspectives, acknowledging that the most relevant demands and resources may differ between caregivers. Exploratory analyses will examine whether items can be statistically grouped into broader demand and resource constructs; where supported by the data, composite variables will be created and analyzed accordingly.

**Mediator: Momentary PF.** PF will be measured momentarily in both work and caregiving contexts using three items adapted to reflect state-level processes: “*I let my thoughts and feelings come and go without controlling them*” (open up), “*I stay focused on what matters and engage in it*” (be present), and “*I act like the sort of person I want to be*” (do what matters). These items represent core components of PF and will be used to examine whether flexibility mediates the relationships between demands/resources and cross-domain outcomes. These items were created by the research team informed by validated instruments [73], clinical ACT expertise, and adjusted to fit the ESM approach (e.g., short, applicable everyday life) [84].

**Cross-Domain Outcomes (dependent).** We will assess positive and negative affect to gain a well-rounded understanding of caregiving-related well-being. Negative affect is measured using items such as: “*I feel anxious*,” “*I feel irritated*,” “*I feel sad*,” “*I feel tense*,” “*I feel guilty*,” “*I feel tired,”* and “*I feel lonely*.” Positive affect is assessed through statements like: “*I feel cheerful*,” “*I feel relaxed*,” “*I feel enthusiastic*,” and “*I feel satisfied*,” based on prior ESM studies in caregivers [85,86]. As an outcome in the work context, work engagement will be assessed using three momentary items: *“I feel energetic,” “I am proud of myself for doing this,”* and *“I am fully involved in doing this.*” These items are derived from the short version of the Utrecht Work Engagement Scale and reflect its three core dimensions: vigor (energy), dedication (pride and involvement), and absorption/positive affective involvement [87]. The items were selected and slightly adapted for momentary assessment to ensure suitability for repeated, real-time measurement in an ESM design, consistent with prior ESM research operationalizing work engagement at the state level. These outcomes will be modelled as dependent variables in analyses testing across-domain effects. Table 2 shows an overview of all variables.

#### Evening questionnaire.

The daily evening questionnaire (see Table 3) will be administered once per day as the final assessment of the day. The purpose of the evening assessment is to capture daily summaries of work and caregiving demands and resources, daily PF, and daily general well-being, enabling examination of day-level spillover processes in line with the W-HR model [21]. Unless otherwise specified, subjective daily items are rated on a 7-point Likert scale ranging from 1 (*not at all*) to 7 (*very much*). Items assessing the number of hours spent working, providing care, or receiving care services are assessed using slider scales, while the type of care service received is assessed using categorical response options. For the multi-item daily work demand and work resource scales, scores will be computed by averaging the corresponding item responses, with higher scores indicating greater daily work demands or greater daily work resources, respectively. Caregiving indicators with different response formats will be analyzed as described below.

**Table 3:** Evening questionnaire items.

Variable	Role	Related daily item(s)
Caregiving Demand	Predictor	Today, caregiving felt emotionally or physically exhausting.Today, caregiving required a lot of effort or energy.How many hours did you spend caring for your family member with dementia today?
Caregiving Resource	Predictor	Today, I had the help or resources I needed to manage caregiving well. What kind of care service(s) did you receive today? For how many hours did you receive the services today?
Work Demand	Predictor	Today, my work felt demanding and required considerable effort.Today, I experienced time pressure at work.Today, my work required a lot of mental or emotional energy.
Work Resource	Predictor	Today, I had enough support to manage my work effectively.Today, I had enough freedom or control over how I did my work.Today, I felt that my work situation allowed me to deal well with my tasks.
Psychological Flexibility	Mediator	Today, I experienced moments when my mind wandered. • During those moments, I was able to stay focused on what’s important. Today, I experienced moments with unpleasant thoughts or feelings. • During those moments, I was able to let them be without trying to push them away immediately. • During those moments, I was able to step back from them, and not let them take over my actions. Today, there were moments when I felt confused by my thoughts or emotions. • During those moments of confusion, I still felt a stable sense of self deep inside Today, I was aware of what matters to me. Today, I got involved in activities that were meaningful, useful, or important to me.
General well-being	Outcome	Today, I felt cheerful and in a good mood. Today, I felt calm and at ease. Today, I had energy and felt lively. Today, I woke up feeling well-rested and refreshed. Today, my day was filled with things that I found interesting or enjoyable.
Job Satisfaction	Outcome	Today, I felt satisfied with how my workday went

**Daily caregiving demands and resources.** Daily caregiving demands and resources will be assessed to capture caregivers’ overall caregiving experience across the day. These measures reflect the accumulated intensity and supportiveness of caregiving demands and resources experienced throughout the day and are conceptualized as time-varying contextual predictors of daily personal well-being. Specifically, demands will be measured using brief indicators reflecting emotional and physical strain as well as time investment, including: *“Today, caregiving felt emotionally or physically exhausting,” “Today, caregiving required a lot of effort or energy,”* and *“How many hours did you spend caring for your family member with dementia today?”* (slider scale). Together, these indicators are thought to capture the daily load and effort associated with caregiving.

Daily caregiving resources will be assessed using both subjective and objective indicators of support availability. Subjective caregiving resources will be measured by caregivers’ perceived access to help and resources for managing caregiving on that day. Objective caregiving resources will be captured through reports of formal care use, including the type of formal care service received (e.g., day care) and total hours of services. These items include: “*Today, I had the help or resources I needed to manage caregiving well.”*, *“What kind of care service(s) did you receive today?”* and *“For how many hours did you receive the services today?”* (slider scale). Together, these indicators reflect daily access to instrumental and formal support, which constitute key caregiving resources within the W–HR framework [21]. Because these indicators use different response formats and capture distinct aspects of the caregiving context, they will initially be analyzed as separate predictors rather than combined into a single unstandardized composite score. Exploratory analyses may examine whether theoretically and empirically supported composite measures can be constructed.

**Daily work demands and resources.** Daily work demands and resources will be assessed as dynamic predictors of daily well-being among employed caregivers. To ensure adequate reliability while minimizing participant burden, both constructs will be measured using brief daily scales consisting of three items each. The items were adapted from established dimensions of the Job Demands–Resources Questionnaire [83] and reformulated for daily assessment. Daily work demands will be assessed using three items capturing the intensity and effortfulness of work on that day, including: *“Today, my work felt demanding and required considerable effort,” “Today, I experienced time pressure at work,”* and “*Today, my work required a lot of mental or emotional energy.”* Together, these items reflect daily workload, time pressure, and cognitive/emotional effort. In addition, daily work resources will be assessed using three items capturing perceived autonomy and support in the work context, including: *“Today, I had enough support to manage my work effectively,” “Today, I had enough freedom or control over how I did my work,”* and *“Today, I felt that my work situation allowed me to deal well with my tasks.”* These items reflect daily access to social/instrumental support and autonomy at work.

**Daily PF.** Daily PF will be measured as a mediating variable in the evening assessment. Daily PF will be assessed using six items adapted from the Psy-Flex questionnaire [73] to capture the core components of PF at the daily level, including attentional focus, acceptance of difficult thoughts and emotions, cognitive defusion, sense of self, values-based action, and present-moment engagement. Conditional introductory or routing questions will be included where appropriate to contextualize the assessment. An example item is: “*Today, I was aware of what matters to me.”* (see Table 3 for full list).

**Daily general well-being.** Daily general well-being will be assessed as the primary day-level outcome. It reflects caregivers’ overall affective, energy, and vitality-related resource state resulting from that day’s experiences across domains. Specifically, five positively worded items adapted from the WHO-5 Well-Being Index will be used, assessing feelings of cheerfulness, calmness, energy, restfulness, and interest or enjoyment during the day [71] (*e.g., “Today, I felt cheerful and in a good mood”*). Items are rated on a 7-point Likert scale ranging from 1 (not at all) to 7 (very much), and a daily well-being score will be computed by averaging item responses.

Although these items are not anchored to a specific activity context, they are conceptualized as indicators of daily general well-being. From this perspective, work and caregiving experiences earlier in the day are expected to influence end-of-day general well-being through cumulative effects on energy, mood, and recovery, making daily general well-being an appropriate outcome for examining caregiving and work spillover at the day level.

**Daily work-related well-being (daily job satisfaction).** Daily work-related well-being will be assessed as an attitudinal work outcome, reflecting caregivers’ overall subjective evaluation of their functioning in the work domain on a given day. In line with the W-HR model, attitudinal outcomes capture employees’ beliefs and feelings about their work role and represent a key indicator of positive work functioning [21]. Daily work-related well-being will be measured using a single global daily item *(“Today, I felt satisfied with how my workday went”*), capturing an overall evaluative appraisal of the workday. This item reflects the integration of affective and evaluative experiences across the day into a global judgment. Single-item measures are commonly used in experience sampling research because the repeated nature of daily assessments requires minimizing participant burden, and multi-item constructs are often not feasible or necessary for concrete, context-specific evaluations [88]. In the present study, daily work-related well-being is conceptualized as a global attitudinal evaluation of the workday, for which a single-item measure appears suitable.

#### ESM debriefing.

At the end of the 14-day ESM period, participants will take part in a structured debriefing procedure designed to conclude study participation in a supportive and transparent manner, monitor participants’ well-being, and gather feedback on experience of the ESM procedure. The debriefing consists of (1) a personalized feedback report and (2) an optional semi-structured debriefing call. The feedback process will focus on a limited number of key themes, use clear and supportive visualizations, and encourage participants’ reflections through open-ended questions [89]. This combined approach aims to ensure data quality, participant support, and meaningful engagement with the ESM feedback.

After completion of the ESM period, participants receive a personalized PDF summary of their ESM data by email. This summary contains descriptive visualizations (e.g., pie charts and time-series graphs) showing participants’ recorded activities, positive affect during caregiving activities, and stress across the 14-day period. Participants are explicitly informed that this summary is descriptive and non-clinical, is not intended for diagnostic or therapeutic purposes, and that there are no “good” or “bad” patterns.

Participants are then invited to take part in an individual debriefing call (approximately 10 minutes) with a trained member of the research team. At the start of the call, voluntary participation is reaffirmed, and participants are reminded of their right to withdraw their data prior to anonymization. During the call, the researcher guides the participant through their personal data summary and invites reflection using scripted prompts (e.g., *When you look at the activity that takes up the largest proportion, does this reflect how you usually spend your time?)*. The researcher explicitly emphasizes that their role is to guide reflection rather than provide clinical interpretation, and that the patterns shown reflect daily experiences rather than evaluations. Following the data review, participants are asked to provide structured feedback on the usability and burden of the m-Path app [90], using Likert-type items administered verbally during the call. Participants indicate their agreement (1 = strongly disagree to 7 = strongly agree) with statements covering: 1) perceived ease of use *(e.g., “I could read the text on the m-Path screen well”; “It was easy to operate the m-Path app during the study”),* 2) instructions and facilitating conditions *(e.g., “The briefing session about using m-Path was clear and sufficient”; “The instructions helped me feel confident using the app independently”),* 3) system burden and feasibility *(e.g., “The frequency of the beeps was manageable in my daily life”; “The timing of the beeps generally fit well with my daily routine”),* 4) question clarity *(e.g., “The questions in the diary were clear and easy to understand”).*

Participants are also given the opportunity to provide open-ended feedback, including responses to the optional questions (e.g., *“Is there anything you discovered during these two weeks that helps you balance work and caregiving?”).* All debriefing interactions are conducted by members of the research team with a background in psychology and experience working with family caregivers of people with dementia. If participants express emotional distress or increased burden during the debriefing, the researcher responds supportively and, if appropriate, encourages contact with certified mental health services. The debriefing concludes with administrative closure, including confirmation of compensation and information about data processing and confidentiality in accordance with GDPR regulations.

#### Incentives for study participation.

To acknowledge participants’ time and effort, incentives will be provided in the form of gift vouchers. All participants who provide informed consent will receive a €10 voucher, regardless of whether they complete the study. To encourage compliance with the daily diary protocol, participants who complete at least 75% of the ESM assessments will receive an additional €10 voucher. The use of vouchers is considered proportionate to the time burden (~7.6–7.8 hours over two weeks) and ensures that participants feel appreciated for their contribution, while avoiding undue inducement.

### Bias

Several steps will be taken to minimize potential sources of bias. Selection bias will be reduced by recruiting participants through multiple channels, including memory clinics, caregiver networks and self-referral channels, and community outreach, to increase representativeness of the caregiver population [91]. Information bias will be minimized by using intensive longitudinal sampling, which reduces recall bias and captures experiences in real time [92]. Attrition bias will be addressed by providing clear instructions, regular check-ins, and incentives for high compliance [93]. Potential confounding will be addressed through a priori, hypothesis-specific covariate adjustment where theoretically justified. Despite these measures, limitations such as the modest sample size and reliance on self-report remain, which may affect generalizability.

### Statistical analysis plan

Analyses will be conducted using R statistical software (version 4.5.1; R Foundation for Statistical Computing, Vienna, Austria) [94]. Descriptive statistics (means, standard deviations, ranges, and frequencies) will be computed for baseline demographic variables, baseline contextual characteristics, and raw momentary and daily variables. Person-mean centering will subsequently be applied to time-varying predictors, including time-varying mediators where applicable, to isolate within-person associations in the multilevel analyses. Baseline measures will primarily be used for sample description and exploratory analyses and may additionally be considered as covariates where theoretically justified.

Prior to hypothesis testing, the reliability and measurement quality of multi-item momentary and daily scales will be evaluated, with particular attention to within-person reliability for repeatedly assessed constructs [95]. Measurement quality will further be evaluated using the ESM-Q tool [84]. Moreover, intraclass correlation coefficients will be calculated to partition variance into within-person and between-person components and to justify the use of multilevel modeling.

#### Handling of missing data.

Compliance rates will be calculated, and patterns of missing data will be examined descriptively across participants, assessment times, and relevant work and caregiving contexts. Missing responses are expected in ESM studies because of the intensive nature of repeated assessments and variation in participants’ daily circumstances [82].

Longitudinal mixed-effects models can accommodate unbalanced numbers of repeated observations and use available outcome data without requiring complete observations at every measurement occasion [96]. Therefore, routine multiple imputation of missing momentary observations is not planned. However, the appropriateness of this approach depends on the nature of the missingness, and missing-data patterns will therefore be examined rather than assumed to be ignorable [97].

For the primary analyses, participants with a compliance rate below 33% of scheduled momentary assessments will be excluded, as specified in the study’s analytical protocol, because very low numbers of repeated observations may provide insufficient within-person information for the planned analyses. For lagged analyses, only observations for which the required consecutive assessments are available will contribute to the corresponding model.

If descriptive inspection or auxiliary analyses indicate that missingness is systematically related to assessment timing, work or caregiving context, participant characteristics, or previously observed responses, sensitivity analyses will be considered to evaluate the robustness of the findings. Where warranted, these may include alternative missing-data approaches that account for the multilevel and temporal structure of intensive longitudinal data [97]. All exclusions, compliance rates, and missing-data patterns will be reported transparently.

#### Primary analyses: Momentary-level spillover.

Momentary hypotheses will be tested using multilevel mixed-effects models to account for the hierarchical data structure, with repeated momentary observations (Level 1) nested within participants (Level 2) [98]. All models will include random intercepts for participants. Random slopes for within-person predictors will be considered when theoretically justified and supported by the data. Given the modest Level-2 sample size, random-effects structures will be kept parsimonious to avoid overparameterization and estimation problems, with any model simplification reported transparently [99,100]. Model specification will be guided by the hypothesized fixed effects, theoretical justification for random effects, model convergence, and the need to avoid overparameterization given the Level-2 sample size. Where nested models differing in fixed-effects structure are formally compared, maximum-likelihood estimation will be used [101]. Prior to model estimation, the internal consistency and within-person reliability of the multi-item momentary composites will be evaluated; if reliability proves insufficient, sensitivity analyses using separate items will be conducted [95]. For the cross-domain lagged analyses, valid lagged pairs will be defined according to the domain reported at consecutive assessments: work-to-caregiving analyses will include pairs in which a work-context assessment at t − 1 is followed by a caregiving-context assessment at t, whereas caregiving-to-work analyses will include the reverse sequence. The distribution of elapsed time between valid consecutive assessments will be examined and reported.

To test work-to-caregiving spillover, momentary work-related demands and work resources assessed at time *t* − *1* will be used to predict caregiving-related well-being at time *t.* Work-related demands will be operationalized through specific indicators of work pressure and workload, while work-related resources will be represented by perceived social support and autonomy during work episodes. To test caregiving-to-work spillover, momentary caregiving-related demands and resources assessed at time *t* − *1* will be used to predict work-related well-being at time *t.* Caregiving-related demands will be operationalized through indicators such as caregiving pressure and caregiving workload, whereas caregiving-related resources will reflect perceived support and autonomy within caregiving episodes. Primary analyses will first examine these specific indicators as separate predictors to preserve the contextual and potentially person-specific nature of demands and resources. Exploratory analyses will subsequently examine whether these indicators can be statistically grouped into broader demand and resource composites. Where supported by factor structure and reliability analyses, composite variables may be created for secondary analyses. Work-related well-being will be computed as the mean of the three work engagement items [87].

PF will be included as a momentary mediator, consistent with the W-HR model, in which volatile personal resources are conceptualized as mediating mechanisms linking contextual demands and resources to subsequent outcomes [21]. It will be operationalized as the mean of the three momentary PF items. For the momentary mediation analyses, demands and resources assessed at time t − 1 will be modeled as predictors of PF at time t, which will in turn be modeled as a mediator of domain-specific well-being at time t. Because PF and well-being are assessed within the same momentary assessment, these models will be interpreted as tests of indirect associations rather than as evidence of a fully temporally ordered causal mediation process [102].

#### Daily-level analyses.

Daily-level hypotheses will be examined using multilevel mixed-effects models, with daily observations (Level 1) nested within participants (Level 2) [98]. This approach allows the examination of within-person, day-to-day fluctuations in demands, resources, and well-being while accounting for between-person differences in average levels of these constructs. Daily time-varying predictors will be person-mean centered to isolate day-specific deviations from individuals’ typical levels, while daily outcomes will be modeled as repeated Level-1 variables [103]. This analytic strategy is appropriate for testing daily spillover processes and avoids conflating within-person and between-person effects [104].

To test the hypothesized day-to-day associations between work and caregiving experiences and subsequent well-being, demands and resources assessed on day d − 1 will be used to predict outcomes on the subsequent day (day d). Daily caregiving demands and resources on day d − 1 will be used to predict daily general well-being and daily work-related well-being on day d. Similarly, daily work demands and resources on day d − 1 will be used to predict daily general well-being on day d. Daily work demands and resources may additionally be examined as predictors of subsequent daily work-related well-being to characterize within-domain associations. Daily PF will be included as a day-level mediator in all daily models. In line with the W-HR model, daily PF is expected to function as a volatile personal resource through which daily demands and resources influence well-being outcomes. Specifically, higher daily demands on day d − 1 are expected to be associated with lower PF on day d, whereas higher daily resources on day d − 1 are expected to be associated with higher PF on day d, which in turn will predict better daily general well-being and higher daily work-related well-being on day d. Mediation effects will be tested within the multilevel framework using appropriate longitudinal mediation procedures for daily diary data [102]. For the mediation analyses, demands and resources assessed on day d − 1 will be modeled as predictors of PF on day d, which will in turn be modeled as a mediator of well-being on day d. Thus, the predictor will be temporally lagged relative to the mediator and outcome, whereas PF and well-being will be assessed within the same day. Accordingly, these analyses will be interpreted as tests of indirect associations rather than as evidence of a fully temporally ordered causal mediation process.

#### Exploratory analyses.

Exploratory analyses will be conducted to examine how baseline individual and contextual characteristics relate to participants’ average levels of daily demands, resources, and well-being across the study period. Specifically, associations will be explored between baseline measures of PF, caregiving context characteristics (e.g., caregiver burden and positive aspects assessed with the CRA), work context characteristics (assessed with the COPSOQ-III), work–home conflict and enrichment (assessed with the SWING), and neuropsychiatric symptom severity and caregiver distress of the person with dementia (assessed with the NPI-Q), and person-level mean scores of state demands, resources, and well-being derived from the repeated assessments.

Potential confounding variables will be identified prior to conducting the corresponding statistical analyses, based on existing evidence, the theoretical framework, and their relevance to the predictor and outcome examined in each model, rather than selected solely on the basis of statistical significance. Candidate covariates include caregiver age and gender, weekly working hours and work arrangement, weekly caregiving hours, relationship to and cohabitation with the person with dementia, availability of additional formal or informal caregiving support, neuropsychiatric symptom severity and associated caregiver distress, baseline caregiver well-being, and baseline PF. Characteristics of the person with dementia, including dementia type and time since symptom onset or diagnosis, may additionally be considered when theoretically relevant to the association under investigation.

Not all measured demographic and contextual characteristics will be included simultaneously in the models. Given the modest Level-2 sample size and the risk of overparameterization, covariate adjustment will be parsimonious and hypothesis-specific. Variables collected primarily for sample characterization, or variables without a plausible confounding relationship with the predictor and outcome of a particular model, will not routinely be included as covariates. Baseline instruments assessing broader constructs, such as the COPSOQ-III, CRA, and SWING, will primarily be used for descriptive characterization and exploratory analyses rather than automatically entered as covariates. Any additional covariate or sensitivity analyses will be clearly identified as exploratory.

Given the limited sample size, these analyses are intended to be descriptive and hypothesis-generating rather than confirmatory. All exploratory analyses will be clearly identified as such, and their results will be interpreted cautiously.

#### Post-hoc analyses.

If unexpected findings arise, post-hoc analyses will be conducted and explicitly labelled as exploratory. To control for inflated Type I error due to multiple testing, multiple-comparison adjustments will be applied using the Benjamini–Hochberg false discovery rate procedure where appropriate [105].

#### Power considerations and expected effect sizes.

In intensive longitudinal research, statistical power depends not only on the number of participants but also on the number of repeated observations contributed by each participant and the amount of within-person variability captured over time [65]. Experience sampling designs are particularly valuable for investigating dynamic processes as they unfold in everyday life, allowing researchers to examine within-person associations and temporal relationships between variables across repeated assessments [63].

The primary analytical focus of the present study is on within-person processes, including lagged spillover effects between work and caregiving domains and the potential mediating role of PF in these associations. Given the intensive repeated-measures design and previous ESM studies on family caregivers of people with dementia [51], the study is expected to provide rich information regarding these day-to-day dynamics. However, more complex effects, such as indirect effects in multilevel mediation models and between-person differences, generally require larger Level-2 sample sizes and are more sensitive to sampling variability [67]. Therefore, mediation analyses and between-person effects will be interpreted with appropriate caution and considered exploratory.

Overall, the study is expected to generate valuable insights into the dynamic mechanisms underlying well-being and PF in employed family caregivers of people with dementia, while providing an empirical basis for future confirmatory and replication studies. The detailed reporting of the sampling design, measures, and analytical approach is intended to facilitate replication of the DAILY-FLEX protocol in other caregiver samples. Where comparable protocols and measures are used, future studies may enable harmonization or pooling of compatible datasets, providing greater statistical power to examine complex effects that cannot be estimated precisely within a single modest-sized ESM sample.

### Safety protocol

Based on earlier ESM studies in comparable populations, the procedure is considered acceptable, and we do not expect any serious adverse events [51]. The present study poses minimal risk to participants, but several safeguards have been implemented to ensure safety and well-being. Participants may withdraw at any time without providing a reason, and they can skip individual assessments if they feel burdened. The estimated study burden (baseline questionnaire, daily diary entries, and debriefing) has been clearly communicated in the Participant Information Sheet. To monitor well-being, the research team will conduct check-ins on day 2 and day 7 of the ESM period. Research team members involved in participant contact have a background in psychology and follow the study safety procedures if participants report distress, including directing participants to appropriate professional services where needed. Technical support will be available throughout, and participants without a smartphone will be provided with a study device and training. Data will be pseudonymized during collection, anonymized before analysis, and securely stored, ensuring confidentiality and GDPR-compliance. These measures safeguard participant rights, minimize risk, and ensure the ethical conduct of the study.

### Limitations

The main limitations of this study include the modest sample size, which may restrict the detection of small between-person effects and limit generalizability. In addition, the reliance on self-report measures introduces potential reporting bias, although the use of ILS helps reduce recall bias. Another limitation is the possibility of selection bias, as participants who consent to daily digital assessments may represent a subgroup of caregivers with greater technological literacy or interest in research participation.

### Strengths

Key strengths of the study are its innovative use of the ESM to capture real-time fluctuations in work and caregiving demands and resources, as well as the inclusion of PF as a theoretically and clinically relevant personal resource factor. Input from experts by experience (Client Panel), ACT clinicians, and the ESM expert group ensures that the study design and measures are both scientifically rigorous and directly informed by end-users’ perspectives. The study contributes novel insights into the work–caregiving interplay of dementia, with potential to guide tailored interventions and inform policy.

### Ethical considerations

This study has received ethical approval from the Faculty of Health, Medicine and Life Sciences Research Ethics Committee at Maastricht University (Approval number: FHML-REC/2025/060). It will be conducted in accordance with the Declaration of Helsinki and the GDPR.

#### Informed consent and voluntary participation.

All participants will be provided with a detailed Participant Information Sheet explaining the study objectives, procedures, potential risks, and benefits. Written informed consent will be obtained digitally via REDCap, a secure web-based system that uses separate tick-boxes for different aspects of consent (e.g., participation, data use, optional data re-use). Participation is entirely voluntary, and participants may withdraw at any time without negative consequences. If withdrawal occurs before data anonymization, all collected data will be deleted upon request. Once data are anonymized and integrated, deletion will no longer be possible, which will be clearly communicated in the information sheet.

#### Confidentiality and data management.

All study data will be collected digitally using GDPR-compliant platforms (Castor EDC and REDCap) and the m-Path smartphone app for daily ESM assessments. Identifiable data (e.g., names and contact details) will be stored separately from research data and accessible only to authorized team members. ESM data will be encrypted during transmission, pseudonymized upon collection, and anonymized before analysis. Anonymized datasets will be securely archived on Maastricht University’s High-performance Networked Attached Storage system for a minimum of 10 years, in accordance with institutional data policies. Access to raw data will be restricted, and data quality will be monitored through regular compliance checks and automated validation procedures (e.g., range checks and missing-data monitoring). Anonymized data retained for future research will only be used for secondary or collaborative research when participants have explicitly consented to such reuse and when the proposed use is consistent with the ethical approval, institutional policies, and applicable data-protection requirements.

#### Burden and potential risks.

The estimated time burden is approximately 7.6–7.8 hours over two weeks, consisting of a baseline questionnaire (~45–55 minutes), daily diary assessments (~29 minutes per day), and a short debriefing (~10 minutes). The main potential risk is increased emotional burden due to repeated self-reflection on caregiving challenges. To minimize this, participants are informed that missing entries is acceptable, items can be skipped, and they may withdraw at any time. Check-ins on day 2 and day 7 will monitor participant well-being.

#### Future use of data.

Consent for the re-use of anonymized data in future research will be explicitly obtained as part of the informed consent process. For participants who provide this consent, anonymized data may be made available for scientifically appropriate secondary analyses and collaborative research. The study is designed to support future replication of the DAILY-FLEX protocol, and researchers conducting comparable intensive longitudinal studies may be able to harmonize or pool compatible datasets with DAILY-FLEX data. Such collaborative use could increase statistical power for examining complex within-person, mediation, and between-person effects that individual ESM studies with modest participant samples may not estimate precisely. Any future sharing or pooling of participant-level data will remain subject to appropriate privacy safeguards and assessment of disclosure risk.

#### Reporting and dissemination.

Results of this study will be disseminated through multiple channels to ensure accessibility for scientific, clinical, and public audiences. Findings will be published in peer-reviewed, open-access journals and presented at international conferences in the fields of dementia care, caregiving, and contextual behavioral science. Interim results and protocol amendments will be documented on the OSF [60]. In addition, lay summaries will be shared with study participants, caregiver organizations, and ACL through newsletters, social media, and community events. Feedback from the Client Panel of ACL will be incorporated into dissemination materials to ensure clarity and relevance for people with dementia and their families.

## Supporting information

S1 Appendix Momentary ESM items. Momentary experience sampling items administered during the 14-day DAILY-FLEX study.
(DOCX)

S2 Appendix Evening questionnaire. Daily evening questionnaire administered during the 14-day DAILY-FLEX study.
(DOCX)
