# Supplementary material for: Psychological flexibility in the work-caregiving interplay of employed family caregivers of people with dementia (DAILY-FLEX): Protocol of an experience sampling study
Source: PLoS One. 2026 Oct 30;21(10):e0361760. doi: 10.1371/journal.pone.0361760 (PMC13722313; doi:10.1371/journal.pone.0361760)
Supplement: Supplementary file 1 [file pone.0361760.s001.docx]

**S1 Appendix. DAILY-FLEX momentary ESM questionnaire**

*Items marked as branching questions were displayed only when participants selected the corresponding triggering response. Unless otherwise indicated, all remaining items were presented at each ESM assessment.*

| **Concept** | **Item** | **Rating scale** |
| --- | --- | --- |
| Positive affect | I feel cheerful  I feel relaxed  I feel enthusiastic  I feel satisfied | 7-point Likert scale (1 = “Not at all”; 7 = “Very much”) |
| Negative affect | I feel anxious  I feel irritated  I feel sad  I feel tense  I feel guilty  I feel lonely  I feel tired | 7-point Likert scale (1 = “Not at all”; 7 = “Very much”) |
| Stress | I feel stressed | 7-point Likert scale (1 = “Not at all”; 7 = “Very much”) |
| Location | Where am I? | At home, At work, Home office, Health care setting, Friend’s/family place, Public setting, Transport, School/University, Somewhere else |
| Social Company | Who am I with? | Family member with dementia , Other family member (s), Friend (s), Supervisor/ manager, Colleague, Health care professional, Acquaintances, Strangers, Other, Nobody |
| Sense of connection | Branching question if with person with dementia:  I feel connected to my family member with dementia | 7-point Likert scale (1 = “Not at all”; 7 = “Very much”) |
| Social appreciation | Branching if any answer except “No body”:  I like being in this company  Branching if “No body”:  I like being by myself | 7-point Likert scale (1 = “Not at all”; 7 = “Very much”) |
| Activity | What am I doing?(just before the alert) | Caregiving (family member with dementia), Caregiving (other,) Household, Work, Study, Self-care, In conversation, Physical activity, Leisure, Resting/relaxing, Something else |
| Caregiving activity | Branching if caregiving (family member with dementia):  What kind of tasks you are performing for your family member with dementia? | Bathing and grooming, dressing, toilet use, mobility, Preparing meals, helping with eating, supervising, medication management, doctor appointments, Providing transportation, other (specify) |
| Doing activity with the partner | I am doing this with my family member with dementia | Yes/No  7-point Likert scale (1 = “Not at all”; 7 = “Very much”)  We use external support , We use technology/ objects, We use reminders/ lists, We shorten/ simplify/ adapt the activity, We adapt the environment, We change our roles in the activity , We accept mistakes, I take over and do the activity, Other, We change nothing  7-point Likert scale (1 = “Not at all”; 7 = “Very much”) |
| Adoption of activity | Branching if yes:  The activity is challenging due to dementia  Branching if > 1 – multiple choice item:  To be able to participate, ….  Branching if any answer except “I take over” or “we change nothing”:  This supports our participation well |  |
| Activity enjoyment | I enjoy doing this | 7-point Likert scale (1 = “Not at all”; 7 = “Very much”) |
| Self-efficacy | I can do this well | 7-point Likert scale (1 = “Not at all”; 7 = “Very much”) |
| Proud of activity | I am proud of myself for doing this. | 7-point Likert scale (1 = “Not at all”; 7 = “Very much”) |
| Psychological Flexibility | I let my thoughts and feelings come and go without controlling them  I stay focused on what matters and engage in it  I act like the sort of person I want to be | 7-point Likert scale (1 = “Not at all”; 7 = “Very much”) |
| Activity workload | I have a lot to do | 7-point Likert scale (1 = “Not at all”; 7 = “Very much”) |
| Activity pressure | I have to work fast | 7-point Likert scale (1 = “Not at all”; 7 = “Very much”) |
| Social support | I feel supported in doing this | 7-point Likert scale (1 = “Not at all”; 7 = “Very much”) |
| Autonomy | I can decide how to carry this out | 7-point Likert scale (1 = “Not at all”; 7 = “Very much”) |
| Work engagement | I feel energetic  I am proud of myself for doing this.  I am fully involved in doing this | 7-point Likert scale (1 = “Not at all”; 7 = “Very much”) |
