# Supplementary material for: Psychological flexibility in the work-caregiving interplay of employed family caregivers of people with dementia (DAILY-FLEX): Protocol of an experience sampling study
Source: PLoS One. 2026 Oct 30;21(10):e0361760. doi: 10.1371/journal.pone.0361760 (PMC13722313; doi:10.1371/journal.pone.0361760)
Supplement: Supplementary file 2 [file pone.0361760.s002.docx]

**S2 Appendix. Evening questionnaire of DAILY-FLEX.**

*Items marked as branching questions were displayed only when participants selected the corresponding triggering response. Unless otherwise indicated, all remaining items were presented once each evening.*

| **Concept** | **Items** | **Rating scale** |
| --- | --- | --- |
| Psychological Flexibility | Today, I experienced moments when my mind wandered.  (if >1): During those moments, I was able to stay focused on what’s important.  Today, I experienced moments with unpleasant thoughts or feelings.  (if >1): During those moments, I was able to let them be without trying to push them away immediately.  (if >1): During those moments, I was able to step back from them, and not let them take over my actions.  Today, there were moments when I felt confused by my thoughts or emotions.  (if >1): During those moments of confusion, I still felt a stable sense of self deep inside  Today, I was aware of what matters to me.  Today, I got involved in activities that were meaningful, useful, or important to me. | 7-point Likert scale (1 = “Not at all”; 7 = “Very much”) |
| General Well-being | Today, I felt cheerful and in a good mood.  Today, I felt calm and at ease.  Today, I had energy and felt lively.  Today, I woke up feeling well-rested and refreshed.  Today, my day was filled with things that I found interesting or enjoyable. | 7-point Likert scale (1 = “Not at all”; 7 = “Very much”) |
| Work Demand  Work Resource | How many hours did you work today?  (if >=1):  Today, my work felt demanding and required considerable effort. Today, I experienced time pressure at work. Today, my work required a lot of mental or emotional energy  Today, I had enough support to manage my work effectively. Today, I had enough freedom or control over how I did my work. Today, I felt that my work situation allowed me to deal well with my tasks. | Slider scale (1–12 hours)  7-point Likert scale (1 = “Not at all”; 7 = “Very much”) |
| Caregiving Demand  Caregiving resource | How many hours did you spend caring for your family member with dementia today?  (if >=1):  Today, caregiving felt emotionally or physically exhausting. Today, caregiving required a lot of effort or energy  Today, I had the help or resources I needed to manage caregiving well.  What kind of care service(s) did you receive today?    For how many hours did you receive the care service (s) today? | Slider scale (1–12 hours)  7-point Likert scale (1 = “Not at all”; 7 = “Very much”)    Housekeeping, Personal care (e.g., bathing, dressing), Nursing, Transportation, Food delivery, walking, Home aid, day care, other, No care services today  Slider scale (1–12 hours) |
| Job satisfaction | Today, I felt satisfied with how my workday went | 7-point Likert scale (1 = “Not at all”; 7 = “Very much”) |
